# Detection of prokaryotic promoters from the genomic distribution of hexanucleotide pairs

**DOI:** 10.1186/1471-2105-7-423

**Published:** 2006-10-02

**Authors:** Pierre-Étienne Jacques, Sébastien Rodrigue, Luc Gaudreau, Jean Goulet, Ryszard Brzezinski

**Affiliations:** 1 Département de biologie, Université de Sherbrooke, Sherbrooke, Québec, Canada; 2 Département d'informatique, Université de Sherbrooke, Sherbrooke, Québec, Canada; 3 Centre d'étude et de valorisation de la diversité microbienne, Université de Sherbrooke, Sherbrooke, Québec, Canada

## Abstract

**Background:**

In bacteria, sigma factors and other transcriptional regulatory proteins recognize DNA patterns upstream of their target genes and interact with RNA polymerase to control transcription. As a consequence of evolution, DNA sequences recognized by transcription factors are thought to be enriched in intergenic regions (IRs) and depleted from coding regions of prokaryotic genomes.

**Results:**

In this work, we report that genomic distribution of transcription factors binding sites is biased towards IRs, and that this bias is conserved amongst bacterial species. We further take advantage of this observation to develop an algorithm that can efficiently identify promoter boxes by a distribution-dependent approach rather than a direct sequence comparison approach. This strategy, which can easily be combined with other methodologies, allowed the identification of promoter sequences in ten species and can be used with any annotated bacterial genome, with results that rival with current methodologies. Experimental validations of predicted promoters also support our approach.

**Conclusion:**

Considering that complete genomic sequences of over 1000 bacteria will soon be available and that little transcriptional information is available for most of them, our algorithm constitutes a promising tool for the prediction of promoter sequences. Importantly, our methodology could also be adapted to identify DNA sequences recognized by other regulatory proteins.

## Background

Adaptation is essential to the survival of any biological organism and requires appropriate transcriptional regulation to modulate gene expression profiles. In prokaryotes, RNA polymerase (RNAP) is responsible for the transcription of all genes. However, promoter recognition is effected by an interchangeable sigma (σ) factor that associates to RNAP, directs the newly formed holoenzyme to a promoter and contributes to transcription initiation [1]. Transcription levels can be further modified by additional regulators (activators and repressors) that affect the recruitment or the activity of RNAP holoenzymes at various promoters [2]. A common feature of σ factors and transcriptional regulators is their ability to recognize specific DNA patterns in order to modulate gene expression. It is presumed that, as a result of evolutionary pressure, these regulatory sequences were selected upstream of some genes or operons and excluded from the rest of the genome.

Bacterial genomes usually encode many σ factors. Of these, the principal σ factor would be responsible for the expression of housekeeping function genes. The remaining σ factors are thought to direct the expression of genes required for specialized functions such as stress responses or sporulation [1]. σ factors can also be classified according to their structural homology to either σ^70 ^or σ^54 ^of *Escherichia coli*. σ^70^-related factors, which constitute the vast majority of known σ factors, are composed of two major DNA-binding domains capable of sensing a certain spacing range when associated to RNAP [3,4]. These σ factors usually recognize two DNA boxes (herein referred as the promoter) of approximately six base pairs (bp) located roughly at 10 and 35 bp upstream of the transcription start site (TSS). Spacing between these two boxes generally ranges from 16 to 20 bp [5]. σ factors similar to σ^54 ^also recognize two DNA boxes in the promoter region. However these elements are located approximately 12 and 24 bp upstream of the TSS. Other major differences between σ^54 ^and σ^70 ^family members are the ability of σ^54 ^to bind DNA in absence of RNAP and the requirement of an isomerization step by an activator to render σ^54^-containing holoenzymes processive.

σ factors can tolerate a variety of mismatches from their consensus sequence. For example, a typical *E. coli *σ^70 ^promoter sequence contains two mismatches within both the -35 and -10 hexanucleotide elements [6]. However, there is generally a direct relationship between promoter strength and the similarity to the corresponding consensus sequence [7]. Variations over three orders of magnitude have been reported in σ^70^-dependent promoter strength in *E. coli *[8]. In some cases, an extended -10 promoter box may be observed and may substitute for the absence of a clear -35 element. Extended -10 promoter boxes were reported to be present in 20% of promoter sequences in *E. coli *[6] and 45% in *Bacillus subtilis *[5].

A variety of techniques have been used to identify TSS and to characterize σ factor-DNA interactions. However, the formal identification of promoters by molecular methods can be tedious and is currently not amenable to genome-wide applications. Consequently, it is important to develop algorithms that can rapidly and accurately evaluate the presence of promoters, without the need for extensive biochemical studies. Current algorithms for promoter detection, typically developed for a specific bacterium, exploit different characteristics of promoter sequences. Some approaches are based on sequence representation or statistical overrepresentation. Other methodologies have also been described for the detection of DNA motifs in sets of regulatory sequences [9-12] or by comparing the upstream regions of orthologous genes from different species [13-17]. A method based on the weaker stability of the DNA double-helix in promoter regions was also recently used to identify promoter regions [18]. However, most of these procedures are not suitable for the identification of precise prokaryotic promoters because of the inherent variability in promoter sequences and because they do not allow variable spacers between two DNA motifs.

Sequence representation strategies designed for promoter identification are usually based on a prior knowledge of some characterized sequences. These algorithms are thus trained to recognize sequences that are similar to a previously defined representation of a promoter. This approach was first used by Galas *et al*. [19]. As reported by Stormo, numerous false positives (FP) are however obtained with this strategy [20]. For example, allowing two mismatches in the σ^70 ^consensus -10 hexanucleotide produces roughly one hit per 30 nucleotides (nt) in the complete genome of *E. coli*. A more accurate representation of DNA-binding motifs consists of position-specific weight matrices (PSWM) [21], and online tools such as Virtual Footprint [22] are available to facilitate their analysis in the context of bacterial gene expression. Nonetheless, searching for full *E. coli *σ^70 ^consensus promoter sequences using more flexible mismatch restrictions offered by PSWM also generates a vast amount of hits [23]. More recently, Huerta and Collado-Vides used a PSWM-derived methodology and detected approximately 15 putative promoters/100 nt in IRs [24]

By adding several constraints such as grouping sequences and filtering with the distance from the start codon, they achieved a sensitivity of 86% with an average of 1.88 putative promoters/100 nt. Several groups have also used general neural networks but no significant improvements have been achieved over the PSWM [25]. Hidden Markov Models (HMM) have also been trained to identify promoter sequences recognized by the principal σ factor in *B. subtilis *[26] and *Campylobacter jejuni *[27]. A learning approach based on a Support Vector Machine (SVM) employing a variant of the mismatch string kernel was also recently described [28]. Importantly, all above-mentioned approaches depend on a previously established or trained description of promoters, and were not designed to function with organisms for which promoter information is insufficient.

Statistical overrepresentation approaches can identify short DNA sequences that are present more frequently in a subset of sequences than what would be expected by chance according to the background distribution. Using such a procedure, Vanet *et al*. have proposed a description of the promoter sequences recognized by the principal σ factor of *Helicobacter pylori *from different sets of IRs [29]. More recently, the MITRA algorithm, which also evaluates the spacing between promoter boxes and the positional bias from the start codon, was applied to 20 bacterial genomes. Four of these genomes generated statistically strong signals possibly corresponding to principal σ factor-dependent promoter sequences, including the ones from *H. pylori *and *B. subtilis *[30]. Using a different approach, the principal σ factor consensus sequence was identified among over-represented motifs in *B. subtilis*, although the methodology was not designed especially for that purpose [31]. The latter study was based on the method of Li *et al*., designed to identify regulatory protein binding sites in *E. coli *[32]. It has been noticed that the *E. coli *σ^70 ^consensus sequence was not identified by this or other approaches, a failure that was attributed to the greater variability of promoter sequences within this organism [32]. A similar method was also unable to distinguish a motif related to the principal σ factor promoters in the complete genome of *Streptomyces coelicolor *[33]. In general, although some statistical approaches had limited success, these methods do not seem appropriate in their current form for the identification of promoter sequences in a variety of organisms.

In this paper, we describe a novel approach based on matrices representing the genomic distribution of hexanucleotide pairs, and designed to predict precise promoter sequences using any annotated prokaryotic genome. This approach can be applied to organisms for which almost no transcriptional data is available, without the need for extensive biochemical characterization. The strategy is based on the observation that, although promoter sequences can vary for every σ factor and according to the GC content of each genome [34], promoters are over-represented in IRs relative to the whole genome. Since this bias appears to be conserved throughout evolution, the characteristic distribution of promoter sequences is thus used to identify promoters in a variety of prokaryotic organisms. Briefly, a score is calculated based on the similarity between a matrix representing the genomic distribution of most promoter sequences reported in the literature and a matrix representing the genomic distribution of a putative promoter sequence. A Z-score is next calculated according to the background. To assess the validity of our method, over 680 characterized promoter sequences from ten genomes were gathered from databases and from the literature, and tested using various statistical indicators. Experimental validations of promoter prediction also supported our approach.

## Results

### Genomic distribution of regulatory sequences is biased and conserved

**Figure 1:**
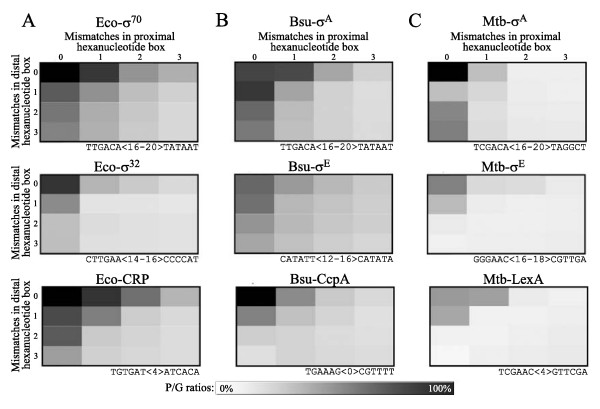
**Genome-derived distribution matrices generated for consensus sequences from transcription factors of three different organisms. **(A) *E. coli*, (B) *B. subtilis*, and (C) *M. tuberculosis*. The name of the transcription factor is identified above each matrix. The mismatch number of each cell is indicated on both sides of matrices. The analyzed consensus sequence is shown under each matrix, along with the allowed spacing range. The first row corresponds to principal σ factors, the second row to alternative σ factors, and the third row to transcriptional regulators.

Transcription initiation is an important step in the regulation of most bacterial cell processes [35,36]. For this reason, it is thought that transcription factor binding sites have been evolutionary selected in some IRs, and generally excluded from the rest of prokaryotic genomes in order to avoid aberrant gene expression. Since regulatory sequences can often tolerate a variety of mismatches with respect to the corresponding DNA motif consensus sequence and still be efficiently bound by their cognate DNA binding protein, these degenerated sequences are also believed to be subjected to a similar selective pressure. Therefore, the genomic distribution of transcription factor consensus DNA binding sequences and close derivatives is expected to be biased towards IRs possibly involved in transcription initiation relative to the whole genome. To test this latter hypothesis, we have analyzed the genomic distribution of consensus sequences representing, respectively, the recognized sequences of a principal σ factor, an alternative σ factor and a transcriptional regulator of three bacterial species (Figure 1 and data not shown). The global distribution of these DNA sequences is reported in "distribution matrices", which consists of tables of dimension 4 × 4 where lines and columns correspond respectively to the number of mismatches in the proximal and distal boxes of a particular DNA sequence. For every matrix cells, the proportion of hits in putative promoter-containing IRs (P) relative to the whole genome (G) was calculated, with respect to a defined spacing range between the two hexanucleotide boxes forming the evaluated sequences. Light colored cells correspond to low P/G ratios while darker cells represent higher frequencies. For example, the genome-derived distribution matrix of the *E. coli *σ^70 ^consensus promoter sequence shows that 65% of genome sequences bearing one mismatch in the -35 element (distal box) but a perfect -10 (proximal) box are localized in P regions relative to the whole genome. At three mismatches in each box (50% degeneracy of the analyzed sequence), about 16% of the hits are found in P regions, which roughly corresponds to the proportion of IRs in the genome. Such sequences are thus considered randomly distributed in the genome. Results presented in Figure 1 thus demonstrate that transcription factor consensus DNA binding sites and related mismatch(es)-containing derivatives tend to be preferentially localized inside IRs. Moreover, the genomic distribution of these sequences shows related patterns in all tested bacterial species (Figure 1).

### Principal σ factor promoter sequences

**Figure 2:**
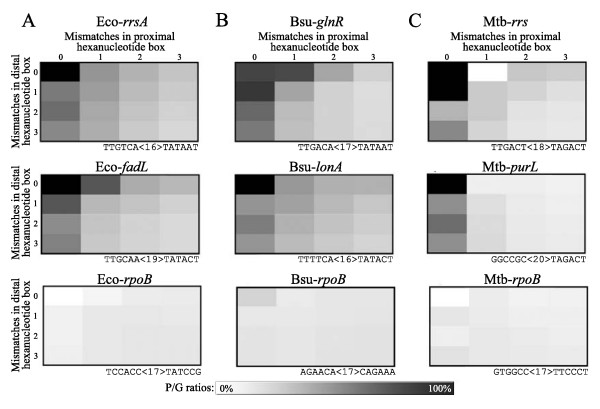
**Specific examples of genome-derived distribution matrices generated for characterized principal σ factor-dependent promoter sequences and non-regulatory sequences from three different organisms**. (A) *E. coli*, (B) *B. subtilis*, and (C) *M. tuberculosis*. Gene names and experimentally identified promoter sequences, as reported in the literature, are indicated above each matrix. The first row corresponds to characterized promoter sequences closely resembling to the proposed consensus. The second row presents experimentally identified promoters containing more mismatches relative to the proposed consensus. The last row shows distribution matrices of hexanucleotide pairs with approximately 3 mismatches position per box, which were extracted from the middle of the *rpoB *coding sequence (*bona fide *non-promoter sequences).

Because principal σ factor promoter sequences are readily available and relatively abundant in many bacterial species, we decided to further explore their genomic distribution. Figure 2 (upper and middle rows) shows genome-derived distribution matrices of experimentally identified promoter sequences from *E. coli*, *B. subtilis *and *M. tuberculosis*. As expected, these matrices contain patterns similar to those observed with the corresponding principal σ factor consensus sequences (Figure 1). Importantly, the genomic distribution of sequences from the coding region of the *rpoB *genes from the same bacterial species did not show any enrichment inside of P regions (Figure 2, last row and data not shown). These results suggest that transcription factor DNA binding sites from various bacterial species have a genomic distribution significantly different from that of non-regulatory sequences.

### Synthetic matrices

From these observations, we hypothesized that a particular genome-derived distribution matrix may appropriately represent several promoters. Hence, it could be possible to calculate a score reflecting the similarity between the distribution matrix of a typical promoter and the genome-derived distribution matrix of any hexanucleotide pair from the same organism. A high score would indicate a strong probability that the tested sequence is also a promoter. However, the best reference matrix should not necessarily be obtained from an existing promoter sequence. In fact, an interpolated matrix could indeed offer more flexibility and be much more effective. We therefore decided to synthetically generate distribution matrices according to the values observed in each cell of the genome-derived distribution matrices for all available experimentally identified principal σ factor-dependent promoter sequences (see Additional file 1: A schematic description of the procedures used in this work). A range of ratios was next determined for each cell, resulting in over 248 million different synthetic matrices (see Additional file 2: Detailed information on the generation of synthetic matrices). Synthetic matrices are thus not affiliated to any hexanucleotide pairs, but are rather produced from the genome-derived distribution matrices of experimentally identified promoters. Moreover, it could be possible to identify synthetic matrices suitable to detect promoter sequences for a specific organism ("organism-specialized matrix"), and perhaps for all bacteria ("general matrix").

### Using distribution matrices to detect promoters

To measure the ability of synthetic matrices to correctly identify promoter sequences, we tested them on 684 characterized principal σ factor dependent promoter sequences from ten bacterial species (Table 1). Complete IRs containing these promoters were extracted from their respective genomes along with 30 nt on each side. These "enlarged IRs" were next scanned using two hexanucleotide windows with respect to the allowed spacing ranges. For each hexanucleotide pair, a genome-derived distribution matrix was generated and used to calculate a score based on its similarity to the synthetic matrix under evaluation (see Additional file 3: Detailed example of score calculation). Different metrics have been tested and the selected one provided the best outcome (data not shown). Since the -10 box has a more important role in transcription [37], the mean of scores sharing the same -10 box was calculated. This latter averaged score had to be greater than the threshold to consider the corresponding sequence as a candidate promoter. Furthermore, since the distance between the TSS and the proximal hexanucleotide box can slightly vary (which may cause the experimental identification of -10 boxes to be inaccurate), signals located at ± 4 nt of an experimentally identified proximal hexanucleotide box were considered as true positives (TP, see Methods). Indicators such as sensitivity, specificity, precision, and performance were calculated for each synthetic matrix [38]. We chose to present the FP rate per 100 nt rather than the precision indicator to facilitate comparison since the precision indicator does not take into account the length of analyzed regions (Table 1). In contrast with other studies [24,39], the analyzed regions were not limited to a maximum length, longer regions producing more FPs.

**Table 1:** Analysis of characterized promoter sequences in ten bacterial genomes.

						**Organism-specialized matrices**			**General matrix**	
**Organism**	**GC**	**Prom.**	**IRs**	**Nt**	**Spacing**	**Sensitivity**	**FP/100 nt**	**Name**	**Sensitivity**	**FP/100 nt**

*E. coli*	50.8%	377	335	117238	16–20	42.4%	1.13	229794169	31.0%	1.09
*B. subtilis*	43.5%	148	142	43446	16–20	56.8%	0.99	113362653	50.0%	0.93
*C. glutamicum*	53.8%	34	33	13572	16–20	29.4%	1.36	223574489	14.7%	1.36
*M. pneumoniae*	40.0%	30	27	7662	15–19	43.3%	1.08	235984178	30.0%	1.07
*M. tuberculosis*	65.6%	28	25	7812	16–20	57.1%	0.74	223574908	50.0%	0.84
*S. coelicolor*	72.1%	17	17	5756	16–20	58.8%	1.42	248361134	47.1%	1.27
*H. pylori*	38.9%	17	16	4725	19–23	47.1%	0.53	91939249	35.3%	0.70
*C. jejuni*	30.5%	14	14	2872	16–20	42.9%	0.84	107109675	35.7%	0.77
*B. japonicum*	64.1%	11	11	4229	16–20	90.9%	0.73	192892765	90.9%	0.97
*S. aureus*	32.9%	8	5	2206	16–20	37.5%	0.59	24624174	37.5%	1.13

For each organism, the GC content, the number of gathered characterized promoter sequences, the number of distinct IRs containing these promoters, the total number of nt in these IRs, and the allowed spacing between hexanucleotide pairs are indicated. The sensitivity and FP rate obtained with the best synthetic matrix (organism-specialized) and the general matrix are also shown.

"Organism-specialized" synthetic matrices (selected on the basis of the performance and sensitivity indicators) gave interesting results for each tested organism (Table 1). For instance, the synthetic matrix #113362653 identified almost 60% of promoters among the set of 148 characterized promoter sequences from *B. subtilis *with approximately one FP/100 nt. Overall, the sensitivity of the best matrix for each organism ranges from 29.4% to 90.9% with 0.53 to 1.42 FP/100 nt (Table 1). Amongst the FP, some could be uncharacterized real promoter sequences. Performance, precision and specificity indicators ranged respectively between 4.6–23.8%, 5.2%-24.4% and 98.6–99.5% (data not shown). Cross-validation tests were also conducted with *E. coli *and *B. subtilis *promoter datasets and matrices very similar to the organism-specialized synthetic matrices were identified. Moreover, the sensitivity and FP rate of these cross-validation matrices were comparable to the organism-specialized matrices obtained using complete datasets, demonstrating the robustness of the approach and suggesting that the various organism-specialized matrices are appropriate (see Additional file 4: Three fold cross-validation results).

**Figure 3:**
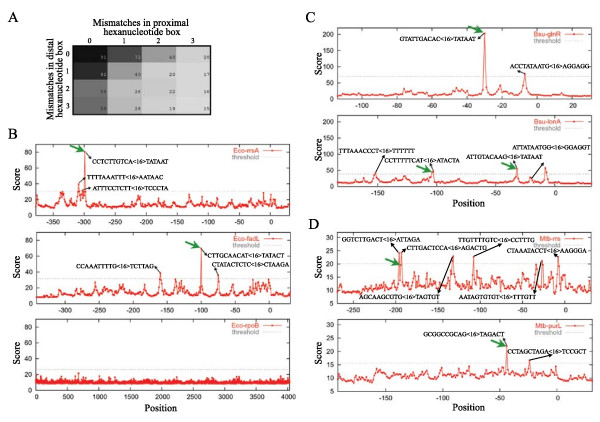
**Graphs of enlarged IRs containing characterized promoter sequences presented in Figure 2**. The green arrow represents the characterized promoter sequence. The start codon of the gene of interest is located at "0" on the X-axis of the enlarged IR. The Y-axis coordinate shows the calculated score. The threshold of each region is shown (dashed grey line). The sequence of all candidate promoters above the threshold is shown (5 merged overlapping -35 boxes from the different allowed spacings along with the shared -10 box). (A) The general synthetic matrix (#45012859) used to calculate the scores presented in the graphs. The name of the gene located downstream of the selected enlarged IR is indicated in each graph. *E. coli *(B), *B. subtilis *(C) and *M. tuberculosis *(D). Scores obtained for the full *E. coli rpoB *coding sequence were also plotted.

Interestingly, several synthetic matrices identified a significant fraction of promoter sequences in all tested genomes. We thus undertook to find a matrix that would be the best compromise for promoter detection in all bacteria. This was achieved by summing up the relative performance indicator of each matrix on all genomes. The best "general" synthetic matrix (#45012859) (Figure 3A and Additional file 3) usually showed a slight decrease in sensitivity relative to organism-specialized synthetic matrices (Table 1). Nonetheless, the sensitivity of this general synthetic matrix ranged from 14.7% to 90.9% with 0.70 to 1.36 FP/100 nt (Table 1), which is comparable to other approaches for an equivalent FP rate (see Additional file 5: Comparison with other bacterial promoter prediction approaches). Furthermore, we have replaced our scoring function by a function based on PSWM. The results obtained with *E. coli *and *B. subtilis *normalized PSWM promoter datasets, showed a significantly decreased sensitivity and a lower FP rate in our design, suggesting that the genomic distribution of promoter sequences is more flexible, and hence more portable between species than current promoter sequence models (see Additional file 6: Comparison with normalized PSWM scoring function).

The scores of all possible hexanucleotide pairs for a specific enlarged IR can be presented in a graph. As an example, Figure 3 shows graphs obtained using the enlarged IRs containing the characterized promoter sequences presented in Figure 2 for *E. coli *(Figure 3B), *B. subtilis *(Figure 3C) and *M. tuberculosis *(Figure 3D). The *rpoB *scanning is also presented for *E. coli *since identical results were obtained using the *B. subtilis *and *M. tuberculosis *sequences (Figure 3 and data not shown). Interestingly, Figure 3C (Bsu-*lonA*) shows one of many examples where a -10 promoter box (consensus TATAAT) is coupled to a ribosome binding site (RBS, consensus AGGAGG).

To assess the significance of our results, the analyses were repeated on two types of shuffled genome sequences. While retaining all the original genome information, the shuffling destroys its structure, which is used by our methodology to identify promoter sequences. The first shuffling procedure was accomplished by repositioning mononucleotides one region (gene or IR) at a time, thus keeping the AT bias intact in each IR [40]. The second shuffling was performed independently of gene annotations, thus dispersing the GC content uniformly through a genome. We surmised that most regulatory sequences, and by extension their genomic distribution, would be affected differently by these procedures. The overall sensitivity obtained with shuffled genomes should thus be decreased. Indeed, genome-derived distribution matrices were completely different if calculated from the real genome or from shuffled genomes (data not shown). As expected, the second shuffling was much more detrimental to the observed sensitivity. For instance, the calculated sensitivity with the general synthetic matrix dropped respectively from 31% (intact genome) to 14% and 0.6% (shuffled genomes) for *E. coli*, and from 50% to, respectively 17% and 0.5% for *B. subtilis*. The same trend has been observed for other genomes (data not shown). Shuffling performed with longer nucleotides blocks than mononucleotide gave intermediate results (data not shown).

### Coupling sequence information to genome distribution

**Figure 4:**
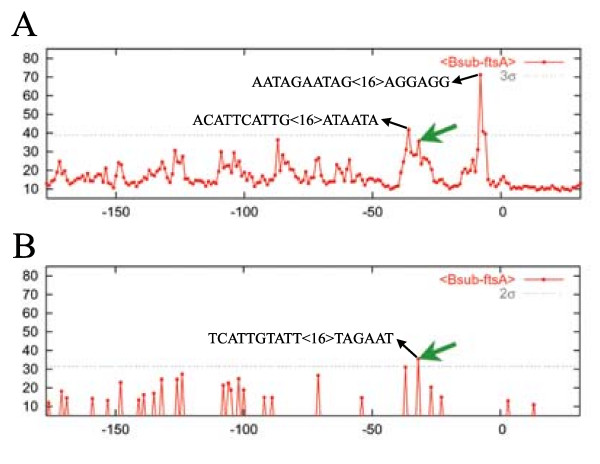
**The application of a simple sequence-dependent filter and the subsequent diminution of the threshold allows the detection of an otherwise missed promoter**. Graphs reporting scores calculated for all hexanucleotide pairs located in the enlarged IR upstream of the *B. subtilis ftsA *gene. (A) No filter applied. (B) Sequence-dependent filtering (3 mismatches and 2 mismatches allowed respectively in the -35 and -10 boxes relative to the *B. subtilis *principal σ factor consensus sequence) followed by the reduction of the region-specific threshold from 3 to 2 standard deviations above of the mean.

For some bacteria, consensus promoter sequences representing the sequences recognized by principal σ factors are well characterized. For example, the consensus promoter sequence for *B. subtilis *and *E. coli *is TTGACA < >TATAAT [5]. We have thus examined the possibility of decreasing the occurrence of FPs by introducing a very simple sequence-dependent filter. Approximately half FPs were eliminated by allowing up to three mismatches in the -35 (distal) box and up to two mismatches in the -10 (proximal) box (the latter being more conserved in these organisms). For instance, a sensitivity of 49% with 0.50 FP/100 nt for the specialized matrix and 44% with 0.44 FP/100 nt for the general synthetic matrix was obtained for *B. subtilis*. Since fewer FPs were observed, the threshold could be lowered, which can improve the sensitivity of the algorithm. For example, the reduction of the IR specific threshold (see Methods) from three to two standard deviations upon sequence filtering gave a sensitivity of 58% with 0.80 FP/100 nt in *B. subtilis *using the general synthetic matrix. Figure 4 illustrates how the application of a filter may allow the identification of an otherwise missed promoter.

### Prediction and biochemical validation of promoters

In addition to the IRs used to evaluate synthetic matrices, we have applied our promoter detection algorithm on the remaining IRs from the ten genomes mentioned in Table 1. Some predicted promoters from *B. subtilis*, *E. coli *and *M. tuberculosis *were chosen for further investigation using a biochemical approach. RNA was extracted from exponentially growing cultures and TSS were determined by primer extension analyses. The seven candidate promoters for which a transcription signal was observed matched the predicted sequence (Table 2). The distance between the observed TSS and the potential -10 box was in all cases either 5 or 6 nt. These results demonstrate that our algorithm represent a valuable tool for the identification of prokaryotic promoter sequences. Complete prediction lists for these three organisms, and others, are available upon request.

**Table 2:** Experimental validation of predicted transcriptional start sites.

**Organism**	**Gene Name**	**Promoter sequence**	**Z-Score**
*E. coli*	*yfgA*	GTGGGCTTTGTCACGAGCACACAGACGGTCTTATACTGTATGA**T**AAC	14.07
*E. coli*	*ygfE*	AAAAGGGCTTGTCTCTTCTCATCAGGGTAGCTATAGTGTCGC**C**CCTT	9.74
*E. coli*	*secE*	ATCATTGCTGAGACAGGCTCTGTTGAGGGCGTATAATCCGAAA**A**GCT	7.75
*E. coli*	*tag*	ATATTATTGTCATTGTATGAAGGATATCGGGCATAGTAGCCCT**G**TAT	5.90
*B. subtilis*	*proB*	AAAAACCTTGACAAGTGTCTTTTTTCTTTGCATAATATAAAA**A**AATC	14.20
*B. subtilis*	*lmrA*	AATTTTTCTTGACAATTGATGATTGAATCAAGATAATAGACC**A**GTCA	11.58
*M. tuberculosis*	*rpsA*	GACCGAGTTTGTCCAGCGTGTACCCGTCGAGTAGCCTCGTCAG**G**TAC	4.69

Bacterial species, gene names, predicted promoter sequences (underlined -35 and -10 boxes), experimentally identified transcriptional start sites (in bold) and the corresponding Z-scores are shown for all tested IRs where a primer extension signal was detected.

## Discussion

In this work, we present evidences suggesting that regulatory sequences and their close derivatives have a biased distribution pattern for IRs that may support transcription initiation. Furthermore, our data support the idea that the preferential location of regulatory sequences is shared between bacterial species. In order to clearly demonstrate the potential of genomic distribution as an indicator of DNA motif function, we have developed an algorithm that can identify a significant fraction of principal σ factor-dependent promoters in any prokaryotic organism, using only a genome annotation and a synthetic matrix (i.e. the general matrix, which was obtained from a training set composed of experimentally identified promoters from ten bacterial species). Promoter predictions were also made and experimentally verified, thus highlighting the potential of our approach for promoter identification in various prokaryotic organisms. Overall, our strategy yielded results similar to those from other studies considering an equivalent amount of FP/100 nt (see Additional file 5: Comparison with other bacterial promoter prediction approaches). However, our algorithm took advantage of a yet unexploited concept, can be used in a wide variety of organisms, required almost no previous knowledge of promoter sequences to be effective, and can be combined with other methodologies. The fact that our general matrix allowed the detection of more promoters in all tested genomes relative to the *E. coli *and *B. subtilis *principal σ factor normalized PSWM (see Additional file 6: Comparison with normalized PSWM scoring function) also supports the idea that genomic distribution of promoter sequences is easier to transfer across organisms with regards to current promoter sequence models.

Although our approach is based on the genomic distribution of hexanucleotide pairs rather than a direct sequence evaluation, it is still important to know the approximate spacing range that is tolerated by a σ factor to efficiently detect the corresponding promoter boxes (data not shown). However, this distance appears to be very similar in most bacteria. The spacing range limitation restrains putative promoter signal contamination by irrelevant hexanucleotide pairs.

An important assumption in our method is that all promoter sequences share a related genomic distribution pattern. However, it is possible that some promoters fall in distinct biological categories or slightly differ between bacterial species. As a consequence, specific matrices could be more adapted to different promoter types. For example, a matrix could be more suitable for promoters containing an extended -10 promoter box. Similarly, very weak promoter sequences could bear an altered distribution pattern when compared to strong promoters.

Another important consideration in our study is the relatively small size of prokaryotic genomes. Since many of these contain only a few million bp, some hexanucleotide pairs are particularly absent from IRs and/or from the whole genome. Therefore, blanks (or 0) in mismatch containing cells can be found in some genome-derived distribution matrices, thus strongly altering the resemblance with the synthetic matrix. Nonetheless, a few promoter sequences containing a blank cell are identified by our approach (see Mtb-*rrs *in Figures 2C and 3C), although most of them are not (data not shown). Since the score is calculated from the mean of hexanucleotide pairs sharing the same proximal box, a blank occurring only at a particular spacing may not be too detrimental to the overall score of a sequence.

An additional possibility to explain our inability to identify some promoters is that, although present in the genome, some mismatch combinations may be relatively rare, and a small variation in absolute numbers may have a significant impact on P/G ratios. In addition, some promoter sequences contain nt triplets corresponding to codons frequently used in translation, which may flatten their distribution bias for IRs. For instance, 19% of the TP and 44% of the FN hexanucleotide pairs of *E. coli *evaluated with the organism-specialized matrix (22% and 41% respectively with the general synthetic matrix) include a triplet which is used as a codon more frequently than average. Thus, almost half of the FN in *E. coli *seem to have an impaired distribution profile because of the inclusion of at least one frequent codon.

In spite of the fact that our algorithm was designed for fully sequenced and annotated genomes, preliminary tests suggest that a genomic distribution calculated from a closely related organism can be used as a reference with similar results (data not shown). Similarly, errors in genomes annotation could theoretically have an impact on the results, albeit we have not observed any significant deterioration of predictions using older versions of the *E. coli *and *B. subtilis *genome annotations (data not shown).

We have shown that combining different detection strategies by applying a very simple sequence-dependent filter to our promoter predictions significantly decreases the FP rate. Since accuracy is a trade-off between sensitivity and the FP rate, this procedure could allow the reduction of the threshold, thus leading to the detection of more TP and increasing the sensitivity. The integration of a more sophisticated sequence-dependent method to our strategy could be used to further reduce the FP rate. Distance filters were also successfully used by other groups to decrease the number of FPs [24,39]. However, this can hardly be justified in biological terms as underlined by Huerta and Collado-Vides [24]. Moreover, such filters may not be suitable for alternative σ factor-dependent promoters or other transcription regulators. We have thus decided not to exploit distance constraints, although it remains possible for an eventual user to determine if a putative promoter is located at an appropriate distance from a gene of interest.

## Conclusion

A simple and intuitive concept about the preferential location of regulatory sequences has allowed the identification of principal σ factor dependent-promoter sequences in the genome of various bacteria. Minimal information about the structure of the searched pattern was only required for our algorithm to detect these promoters. Moreover, it could be possible to predict promoters in species for which little transcriptional information is available using the proposed general matrix. Since a biased distribution pattern also appear to be conserved for alternative σ factors and other regulatory proteins in a variety of prokaryotes, it should be possible to design distribution matrices to identify their corresponding DNA binding sites.

## Methods

### Promoter sequences, corresponding genomes and IRs datasets

Most of the *E. coli *and *B. subtilis *characterized promoter sequence datasets were respectively gathered from EcoCyc version 8.0 [41,42], and DBTBS release 3.1 [43,44], and the literature [5,45]. To circumvent possible errors in promoter datasets, consistency tests against corresponding genomic sequences were performed [38] with the ASAP gene annotation version m54 for *E. coli *K-12 strain MG1655 [46,47], and SubtiList release R16.1 for *B. subtilis *[48,49]. Binding sites that were not unambiguously detected in their corresponding genome were excluded. The complete corresponding IRs, to which 30 nt were added on both sides, were then extracted from each genome. This resulted in 377 characterized *E. coli *σ^70^-dependent promoter sequences from 335 different enlarged IRs, and 148 *B. subtilis *σ^A^-dependent promoter sequences from 142 enlarged IRs. The procedure was also applied to promoter sequences found in the MtbRegList database release 1.1 for *M. tuberculosis *[50,51], and for characterized promoters identified from the literature for *Corynebacterium glutamicum *[52], *M. pneumoniae *[53], *S. coelicolor *[54], *H. pylori *[55-57], *C. jejuni *[58], *B. japonicum *[59-64], and *S. aureus *[65-69] (Table 1). Genome annotations originated from: *M. tuberculosis *H37Rv (TubercuList R6) [70,71], *C. glutamicum *ATCC13032 (NC_003450.3) [72], *M. pneumoniae *M129 (NC_000912.1), *S. coelicolor *A3(2) (NC_003888.3), *H. pylori *26695 (PyloriGene R1.6) [73,74], *C. jejuni *NCTC11168 (NC_002163.1), *B. japonicum *(NC_004463.1), and *S. aureus *Mu50 (NC_002758.2). Since there is no principal σ factor promoter consensus sequence clearly identified for *M. tuberculosis*, promoter sequences were selected as for groups A and B of Table 1 in Gomez and Smith [75] using the MtbRegList database. Similarly, only *S. coelicolor *promoter sequences from Table 1 of Strohl [54] were considered. Datasets are available in Additional file 7.

### Genome-derived distribution matrices

Genomic distributions of hexanucleotide pairs were represented by a ratio of the number of hits in IRs located upstream of a gene (P) to total hits in the whole genome (G). Hits were counted only on the functional strand (on the same strand than the following coding sequence) for all spacings inside the allowed spacing range. Identical hexanucleotide pairs with different spacer length will thus have the same genome-derived distribution matrix provided that their respective spacings are included in the allowed range. The genomic distribution of up to three exclusive mismatches per hexanucleotide was also reported in genome distribution matrices. Ratios at various mismatches combinations were reported in genome-derived distribution matrices of dimension 4 × 4 (Figure1, 2). Columns and rows respectively represent mismatches in the -10 (proximal) and -35 (distal) boxes.

### Synthetic matrices

248 371 200 distribution matrices were generated *in silico *and referred to as "synthetic matrices". To create these, the genome-derived distribution matrices of almost all characterized promoter sequences available were analyzed, and the range of variation in each cell was determined in accordance with the observed ratios. The range and step length was independently established in each cell. Detailed information about synthetic matrices is available in Additional file 2.

### Score calculation

To calculate a score, the genome-derived distribution matrix of a hexanucleotide pair was compared to a synthetic matrix. The analytical approach was inspired by the image processing field and involved four components, each representing the mean of square differences between matrices: R_1 _is calculated on the raw data of the matrices, and R_2 _to R_4 _are respectively calculated on the horizontal, vertical and diagonal directional derivatives of matrices to evaluate the three different slopes of the matrices. Each slope is related to the representation of the genomic distribution of hexanucleotide pairs (proximal and distal boxes). A weight (w) of ¼ is next applied to each component. The final score = 1/(wR_1_+ wR_2_+ wR_3_+ wR_4_). See Additional file 3 for a detailed example of score calculation.

### IR scanning

IR scanning was accomplished by taking the first six most upstream nt of an enlarged IR along with the downstream hexanucleotide window located at the shortest distance within the specified spacing range. A genome-derived distribution matrix was then generated and a score was calculated with the above described score metric. This procedure was repeated for all allowed spacings by moving the downstream hexanucleotide window by one nt. The upstream hexanucleotide was next moved by one nt and the same procedure was repeated until all appropriate hexanucleotide pairs of the region were processed. The mean of values obtained for all hexanucleotide pairs sharing the same proximal box were then plotted on a graph (Figure 3). Using the maximum values instead of the mean gave very similar results (data not shown). Two thresholds were selected. The region threshold (tR) was set at three standard deviations above the mean of all points from a specific IR. The genome threshold (tG) was set at two standard deviations from the mean of all points from all IR of a genome. tR and tG were optimized using *E. coli *and *B. subtilis *promoters data (data not shown). The value of any point had to be higher than both thresholds to be considered as a candidate promoter. All adjacent points above thresholds were combined in one peak and represented by their highest point. The widest peak has 6 points and the mean is 1.25 point per peak. A peak had to be located within 4 nt of an experimentally identified TSS to be considered as a TP. All other points above thresholds were considered as FPs. Points representing characterized hexanucleotide pairs below the highest threshold were considered as FNs, while all other points below this threshold were considered as TNs. According to Tompa *et al*., sensitivity is defined as TP/(TP + FN), specificity as TN/(TN + FP), precision (or positive predictive value) as TP/(TP + FP), and performance as TP/(TP + FN + FP) [38].

### Evaluation of synthetic matrices

The evaluation of over 248 million synthetic matrices on the 625 enlarged IRs (containing 684 characterized promoter sequences from the ten genomes mentioned in Table 1) was performed on the Mammouth Linux cluster of the Université de Sherbrooke (1808 processors, 7.6 Tflops, 5.5 TB of RAM, 160 TB of HD). The performance score for each synthetic matrix was calculated and the best matrix selected as the specific matrix for each genome (Table 1). The general synthetic matrix was selected from the sum of the relative performances of each matrix on each genome following S = (sum_*i*_(perf_*ij*_/maxPerf_*j*_)) where perf_*ij *_represents the performance score of a given matrix in the organism *j*, and maxPerf_*j *_represents the maximum performance score of all matrices in the organism *j*.

### Cross-validation tests

Three-fold cross-validation tests were conducted with 1% of the synthetic matrices randomly chosen from the previously described set. The *E. coli *and *B. subtilis *datasets were randomly divided into three groups, and all possible combinations of two groups were used to select new specialized synthetic matrices. The statistical indicators were next calculated on the remaining group. Results are presented in Additional file 4.

### PSWM scoring function

The scoring function of our method was replaced by a function based on PSWM scores. The rest of the IR scanning procedure remained absolutely identical to the initial design. The promoter datasets of *E. coli *and *B. subtilis *were used to construct PSWMs, which were normalized according to the intergenic ATGC content of the tested genome. Results are presented in Additional file 6.

### Shuffled genome

Two shuffled genomes were created. First, the regions (genes and IRs) were independently shuffled to conserve the possible AT bias of IR [40]. The second type was made on the entire genome so that no bias is kept. Shuffled genomes were next used to calculate genome-derived distribution matrices to assess the same enlarged IRs previously analyzed (data not shown).

### Codon usage evaluation

By definition, a hexanucleotide contains 4 overlapping codons. The mean of the utilization ratio of the eight codons of a hexanucleotide pair was thus compared to the average usage frequency of a codon (15.62/1000 residues for *E. coli*) to evaluate if there is a difference between hexanucleotide pair sequences precisely identified (TP) or missed (FN) (data not shown). Codon usages were taken from the Codon Usage Database [76].

### Biochemical validation of predicted promoters

All IRs for which no promoter sequence is characterized in *B. subtilis*, *E. coli *and *M. tuberculosis*, were analyzed with their respective organism-specialized matrix to predict putative promoters. In order to validate some predicted promoters under the control of the housekeeping σ factor, predictions were selected on the basis of the putative function of their corresponding gene, the Z-score, the loci organization and the promoter sequences. Validation of the *M. tuberculosis *prediction was made on the closely related non-pathogenic *M. bovis *BCG-Russia. *E. coli *K12 ATCC10798 and *B. subtilis *NIG2001 [77] were grown in LB medium. *M. bovis *BCG-Russia was grown in Middlebrook 7H9 medium supplemented with Albumine-Dextrose-Saline, Tween 80 and cycloheximide. All cultures were harvested at an OD_600 _between 0.6 and 0.8 and RNA was extracted using the Ribopure RNA extraction kit (Ambion) or the RNeasy kit (Qiagen). RNA was quantified by spectrophotometry and integrity was verified on formaldehyde denaturing gel. Primer extensions were performed according to standard procedures. Between 30 and 60 μg of RNA were used for each reaction. Extension products were migrated on 5M urea-6% acrylamide sequencing gels along with sequencing reactions. IRs were cloned in pCR2.1-TOPO TA cloning vector (Invitrogen) or pdrive TA cloning vector (Qiagen). Oligonucleotide primers are listed in Additional file 8. Sequencing ladders were produced with the Sequenase 2.0 kit (USB) according to the manufacturer's instructions. Gels were scanned using a Molecular Dynamics Storm 840 Phosphorimager.

## Authors' contributions

PEJ contributed to the basic concept of the study, elaborated the design of the study, performed almost all the analysis, and wrote the manuscript. SR contributed to the design of the study, carried out the biochemical validation, and wrote the manuscript. LG and JG contributed to the design and coordination of the study, and helped writing the manuscript. RB elaborated the basic concept of the study, contributed to the design and coordination of the study, and helped writing the manuscript. All authors read and approved the final manuscript.

## Supplementary Material

Additional file 1 A schematic description of the procedures used in this work. A schematic description of the procedures used in this work. Click here for file

Additional file 2 Detailed information on the generation of synthetic matrices. Minimum and maximum values with step length and number of values for the construction of each cell of the synthetic matrices. Click here for file

Additional file 3 Detailed example of score calculation. Detailed example for each step of score calculation. Click here for file

Additional file 4 Three fold cross-validation results. Cross-validation results for each of the "training" and "testing" phases, along with scores between matrices identified by each training phases and their respective organism-specialized matrix. Click here for file

Additional file 5 Comparison with other bacterial promoter prediction approaches. Comparison between our method and other bacterial promoter prediction approaches. Three variations of the region-specific threshold are shown for organism-specialized matrices. Click here for file

Additional file 6 Comparison with normalized PSWM scoring function. Comparison of our integral method with a method in which the scoring function was replaced by normalized PSWM scores. Click here for file

Additional file 7 Datasets of promoter sequences and enlarged IRs. Fasta format where the title line of each sequence contains the following information: > "Organism name" _ "Gene name" "-35 hexanucleotide" < "Spacing" > "-10 hexanucleotide" _ "Name of the sigma factor when available" _ "Exclusive distance between the 3' base of the -10 hexanucleotide and the first base of the start codon" _ "Length of the complete IR". The 30 nt upstream and downstream each IR are also respectively included. Click here for file

Additional file 8 Primer sequences used for biochemical validations. Primer sequences used for biochemical validations. Click here for file
