# Supplementary material for: Detection of prokaryotic promoters from the genomic distribution of hexanucleotide pairs
Source: BMC Bioinformatics. 2006 Oct 2;7:423. doi: 10.1186/1471-2105-7-423 (PMC1615881; doi:10.1186/1471-2105-7-423)
Supplement: Additional file 1 — A schematic description of the procedures used in this work. A schematic description of the procedures used in this work. [file 1471-2105-7-423-S1.pdf]

Additional file 1 – A schematic description of the procedures used in this work

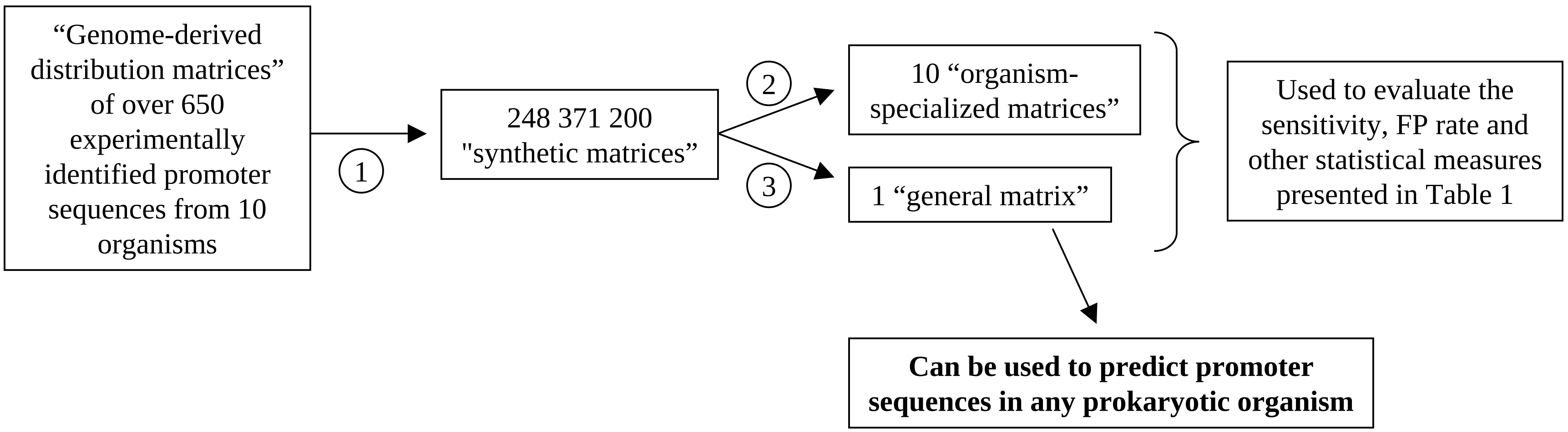

1) The genomic distribution ratios for each cell of the distribution matrices from all available experimentally identified promoters were analyzed. A set of minimum and maximum values, along with step length was determined for each cell (see Additional file 2 for more details), and synthetic matrices were generated form this data to account for every possible cell combinations.

2) Evaluation of all synthetic matrices on the available promoter sequence dataset of each organism. The most effective synthetic distribution matrix at detecting the experimentally identified promoters from a given bacterium is next referred as the corresponding organism-specific matrix.

3) Evaluation of all synthetic matrices on all available promoter sequences. The best performing synthetic distribution matrix is referred as the general matrix, and is capable of identifying promoter in all tested bacterial genomes, and possibly in the genome of any bacteria.
