# Supplementary material for: Detection of prokaryotic promoters from the genomic distribution of hexanucleotide pairs
Source: BMC Bioinformatics. 2006 Oct 2;7:423. doi: 10.1186/1471-2105-7-423 (PMC1615881; doi:10.1186/1471-2105-7-423)
Supplement: Additional file 2 — Detailed information on the generation of synthetic matrices. Minimum and maximum values with step length and number of values for the construction of each cell of the synthetic matrices. [file 1471-2105-7-423-S2.pdf]

Additional file 2 – Detailed information on the generation of synthetic matrices

	0/0	0/1	0/2	0/3	1/0	1/1	1/2	1/3	2/0	2/1	2/2	2/3	3/0	3/1	3/2	3/3
**Minimum_value**	0.900	0.200	0.210	0.163	0.465	0.313	0.200	0.143	0.444	0.264	0.196	0.164	0.459	0.260	0.192	0.154
**Maximum_value**	0.999	0.845	0.396	0.198	0.825	0.436	0.232	0.187	0.678	0.322	0.216	0.164	0.587	0.283	0.192	0.154
**Step_length**	0.011	0.043	0.031	0.035	0.036	0.041	0.032	0.022	0.039	0.029	0.020	0.000	0.032	0.023	0.000	0.000
**Number_of_values**	10	16	7	2	11	4	2	3	7	3	2	1	5	2	1	1
