# Supplementary material for: Detection of prokaryotic promoters from the genomic distribution of hexanucleotide pairs
Source: BMC Bioinformatics. 2006 Oct 2;7:423. doi: 10.1186/1471-2105-7-423 (PMC1615881; doi:10.1186/1471-2105-7-423)
Supplement: Additional file 4 — Three fold cross-validation results. Cross-validation results for each of the "training" and "testing" phases, along with scores between matrices identified by each training phases and their respective organism-specialized matrix. [file 1471-2105-7-423-S4.pdf]

Additional file 4 – Three fold cross-validation results

"Traning" phase

					Organism-specialized matrices		General matrix		
**Organism**	**Promoters**	**Regions**	**Nt**	**Spacing**	**Sensitivity**	**FP/100 nt**	**Name**	**Sensitivity**	**FP/100 nt**
E. coli	**377**	**335**	**117238**	**16-20**	**42.4%**	**1.13**	**229794169**	**31.0%**	**1.09**
Ecoli-1_2	252	252	87198	16-20	40.5%	1.14	203401358	27.8%	1.11
Ecoli-1_3	251	251	90404	16-20	45.8%	1.17	203395079	33.9%	1.14
Ecoli-2_3	251	251	90026	16-20	45.4%	1.15	226912098	33.1%	1.15
B. subtilis	**148**	**142**	**43446**	**16-20**	**56.8%**	**0.99**	**113362653**	**50.0%**	**0.93**
Bsub-1_2	99	99	32359	16-20	59.6%	0.85	173272688	56.6%	0.96
Bsub-1_3	99	99	30648	16-20	55.6%	0.89	222023835	48.5%	0.96
Bsub-2_3	98	98	28705	16-20	55.1%	0.89	20218415	46.9%	0.96

"Testing" phase

					Organism-specialized matrices		General matrix		
**Organism**	**Promoters**	**Regions**	**Nt**	**Spacing**	**Sensitivity**	**FP/100 nt**	**Name**	**Sensitivity**	**FP/100 nt**
Ecoli-3	125	125	46616	16-20	46.4%	1.19	203401358	39.2%	1.17
Ecoli-2	126	126	43410	16-20	38.9%	1.16	203395079	27.0%	1.12
Ecoli-1	126	126	43788	16-20	39.7%	1.20	226912098	28.6%	1.11
Bsub-3	49	49	13497	16-20	42.9%	0.89	173272688	38.8%	0.96
Bsub-2	49	49	15208	16-20	51.0%	0.93	222023835	55.1%	0.96
Bsub-1	50	50	17151	16-20	56.0%	0.91	20218415	58.0%	0.96

Scores between specialized matrices

	**Name**	**229794169**
Ecoli-1_2	**203401358**	504.84
Ecoli-1_3	**203395079**	2379.75
Ecoli-2_3	**226912098**	569.57

	**Name**	**113362653**
Bsub-1_2	**173272688**	106.88
Bsub-1_3	**222023835**	131.73
Bsub-2_3	**20218415**	221.35

Refered to the Figure 3 of the article to get an idea of the magnitude of the scores.
