# Supplementary material for: Detection of prokaryotic promoters from the genomic distribution of hexanucleotide pairs
Source: BMC Bioinformatics. 2006 Oct 2;7:423. doi: 10.1186/1471-2105-7-423 (PMC1615881; doi:10.1186/1471-2105-7-423)
Supplement: Additional file 5 — Comparison with other bacterial promoter prediction approaches. Comparison between our method and other bacterial promoter prediction approaches. Three variations of the region-specific threshold are shown for organism-specialized matrices. [file 1471-2105-7-423-S5.pdf]

Additional file 5 – Comparison with other bacterial promoter prediction approaches

E. coli

Name	Sensitivity	FP/100 nt	Promoters	Regions	Spacing	Method	Comment	Reference
**Genomic Distribution**	97.1%<br>42.4%<br>28.1%<br>31.0%	9.26<br>1.13<br>0.59<br>1.09	377	335	16-20	Organism-specialized matrix<br>General matrix	See discussion.	This work
**Statistical over-representation generating PSWM**	N/D	N/D	N/D	~2500	0-30	Words from 3 to 5 nucleotides were analyzed in the first 300 nt of IRs located upstream of all operons.Statistically significant dimmers with fixed-length spacers were grouped into clusters of related sequences. Putative functions were assigned to these sequence clusters by examining their location in the genome.	No sigma70 consensus sequence was identified. This was attributed to the greater variability of promoters in this organism.	[32]
**PSWM**<br>**PSWM**<br>**Cover**	100%<br>47.7%<br>86.0%	15.10<br>0.87<br>1.88	599	470	13-24	PSWM search limited on 250 nt of each region, cutoff mean-3σ.<br>PSWM search limited on 250 nt of each region, cutoff mean-0.5σ.<br>PSWM with several constraints such as grouping signals and filtering with the distance from the start codon.	Any approach based on PSWMs depends on the availability of an extensive experimental dataset to create a representative description of the As the authors noted, a distance filter is "hard to reconcile with a direct computational modeling of RNAP binding and transcription initiation".	[24]
**MITRA**	N/D	N/D	N/D	589	3-23	The significance of hexanucleotide pairs in the first 310 nt of divergent IRs was estimated by comparaison with those of convergent IRs considering three type of information: the strength score, the dyad score, and the positional score.	No statistically strong signal corresponding to a principal sigma factor-dependent promoter sequence was identified in this organism.	[30]
**NNPP2.2**	31.0%	0.62	671	510	N/D	Neural network trained with 272 *E. coli* promoters. Promoter searches were limited to the first 500 nt of every tested regions. Default cutoff was set to 0.8 and a shift of ± 3 nt was tolerated with respect to the location of the experimentally identified promoter.		[78]
**TLS-NNPP2.2**	30.1%	0.22				Distance filter combined to NNPP2.2. Cutoff set to 0.0269.	See comment on the use of a distance filter in the "Cover" approach.	[39]
**HMM**	N/D	N/D	N/A	N/A	N/A	Same methodology as for *C. jejuni* (see below).	A TATA-box of varying intensity was identified but no periodic signal could be seen in sequence logos of predicted promoters aligned by the model.	[27]
**SVM**	N/A	N/A	450	450	N/D	A kernel with strings of length 5 and 1 mismatch trained with sets of 200 nt each.	The results presentation do not allowed sensitivity and FP rate extraction.	[28]
**DNA stability**	32.0%	1.20	227	227	N/D	The free energy of a 15 nt moving window is calculated on regions of 1000 nt each. If two positive signals (higher than thresholds) are within 25 nt of each other, they were considered as 1 segment. A TP is a segment that overlap the 200 nt region spanning from -150 to +50 from the characterized TSS.	As the authors noted, this "method tries to find a promoter region" rather than identifying precisely promoter boxes.	[18]

B. subtilis

Name	Sensitivity	FP/100 nt	Promoters	Regions	Spacing	Method	Comment	Reference
**Genomic Distribution**	100%<br>84.5%<br>56.8%<br>50.0%	8.42<br>4.29<br>0.99<br>0.93	148	142	16-20	Organism-specialized matrix<br>General matrix	See discussion.	This work
**Statistical over-representation generating PSWM**	82.5%	*0.2%	132	2729	3-30	Methodology based on [32]. Words of 4 and 5 nts. Their PSWM correspondig to consensus promoter sequence (WM1= $N_7TTGAN_{19}TATAATAN_6$) predicted 1141 promoter sequences, including 109 of the 132 contaning a spacing of 17.	*The FP rate was estimated from an expected normal distribution of the data. No actual count of false predictions. Both sensitivity and FP rate would have to be considered for fair comparison.	[31]
**MITRA**	N/D	N/D	N/D	552	3-23	The significance of hexanucleotide pairs in the first 310 nt of divergent IRs was estimated by comparaison with those of convergent IRs considering three type of information: the strength score, the dyad score, and the positional score.	The identified consensus sequence was not used to make predictions.	[30]
**HMM**	70.0%	*0.0	130	N/A	N/D	Trained with 100 nt sequences from approximately -85 to +15 relative to the transcription start site.	*The FP rate was estimated from 1000 random sequences of 100 nt, with respect to the GC% of the genome. Similarly, almost no FPs are produced by our algorithm with the second version of shuffled genomes.	[26]
**DNA stability**	N/A	N/A	89	89	N/D	The free energy of a 15 nt moving window is calculated on regions of 1000 nt each. If two positive signals (higher than thresholds) are within 25 nt of each other, they were considered as 1 segment. A TP is a segment that overlap the 200 nt region spanning from -150 to +50 from the characterized TSS.	As the authors noted, this "method tries to find a promoter region" rather than identifying precisely promoter boxes.	[18]

H. pylori

Name	Sensitivity	FP/100 nt	Promoters	Regions	Spacing	Method	Comment	Reference
**Genomic Distribution**	100%<br>70.6%<br>47.1%<br>35.3%	6.98<br>3.11<br>0.53<br>0.70	17	16	19-23	Organism-specialized matrix<br>General matrix	See discussion.	This work

Name	Sensitivity	FP/100 nt	Promoters	Regions	Spacing	Method	Comment	Reference
**Statistical over-representation**	N/D	N/D	N/D	756, 340 and 30	8-10 to 22-24	These authors used three datasets: a) both strands from all non-convergent IRs; b) divergent IRs only; c) IRs located upstream of ribosomal genes. They were used to identify hexanucleotide pairs present with at most one mismatch in at least 10% of the sequences of the sets.	The most statistically significant motif was used to identified 56 putative sigma 80 promoter sequences. However, none of these predictions correspond to the 17 characterized promoters described in the literature and used in our work.	[29]
**MITRA**	N/D	N/D	N/D	169	3-23	The significance of hexanucleotide pairs in the first 310 nt of divergent IRs was estimated by comparaison with those of convergent IRs considering three type of information: the strength score, the dyad score, and the positional score.	The identified consensus sequence was not used to make predictions.	[30]

C. jejuni

Name	Sensitivity	FP/100 nt	Promoters	Regions	Spacing	Method	Comment	Reference
**Genomic Distribution**	100%<br>71.4%<br>42.9%<br>35.7%	7.90<br>3.13<br>0.84<br>0.77	14	14	16-20	Organism-specialized matrix<br>General matrix	See discussion.	This work
**HMM**	70.3%	*0.0	27	22	N/A	Trained with 175 divergent IRs of 121 nt each. The authors proposed a consensus sequence composed of an AT-rich periodic signal upstream of a classical –10 box (TATAAT sequence). No –35 box was formally identified.	*The FP rate was estimated on sequences generated randomly. Some of the 27 promoters used for sensitivity evaluation have not been detected in the genome.	[27]
**MITRA**	N/D	N/D	N/D	168	3-23	The significance of hexanucleotide pairs in the first 310 nt of divergent IRs was estimated by comparaison with those of convergent IRs considering three type of information: the strength score, the dyad score, and the positional score.	No statistically strong signal corresponding to a principal sigma factor-dependent promoter sequence was identified in this organism.	[30]

M. pneumoniae

Name	Sensitivity	FP/100 nt	Promoters	Regions	Spacing	Method	Comment	Reference
**Genomic Distribution**	100%<br>76.7%<br>43.3%<br>30.0%	8.89<br>3.20<br>1.08<br>1.07	30	27	15-19	Organism-specialized matrix<br>General matrix	See discussion.	This work
**MITRA**	N/D	N/D	N/D	52	3-23	The significance of hexanucleotide pairs in the first 310 nt of divergent IRs was estimated by comparaison with those of convergent IRs considering three type of information: the strength score, the dyad score, and the positional score.	No statistically strong signal corresponding to a principal sigma factor-dependent promoter sequence was identified in this organism.	[30]

S. aureus

Name	Sensitivity	FP/100 nt	Promoters	Regions	Spacing	Method	Comment	Reference
**Genomic Distribution**	87.5%<br>62.5%<br>37.5%<br>37.5%	5.85<br>2.63<br>0.59<br>1.13	8	5	16-20	Organism-specialized matrix<br>General matrix	See discussion.	This work
**HMM**	N/D	N/D	N/A	N/A	N/A	Same methodology as for *C. jejuni* (see above).	A TATA-box of varying intensity but no periodic signal could be seen in sequence logos of predicted promoters aligned by the model (data not shown).	[27]

N/D = Non-determined
N/A = Non-available

References

18 Kanhere A, Bansal M: **A novel method for prokaryotic promoter prediction based on DNA stability**. *BMC Bioinformatics* 2005, **6**:1-10.
24 Huerta AM, Collado-Vides J: **Sigma70 promoters in *Escherichia coli*: specific transcription in dense regions of overlapping promoter-like signals**. *J Mol Biol* 2003, **333**:261-78.
26 Jarmer H, Larsen TS, Krogh A, Saxild HH, Brunak S, Knudsen S: **Sigma A recognition sites in the *Bacillus subtilis* genome**. *Microbiology* 2001, **147**:2417-24.
27 Petersen L, Larsen TS, Ussery DW, On SL, Krogh A: **RpoD promoters in *Campylobacter jejuni* exhibit a strong periodic signal instead of a -35 box**. *J Mol Biol* 2003, **326**:1361-72.
28 Gordon JJ, Towsey MW, Hogan JM, Mathews SA, Timms P: **Improved prediction of bacterial transcription start sites**. *Bioinformatics* 2006, **22**:142-8.
29 Vanet A, Marsan L, Labigne A, Sagot MF: **Inferring regulatory elements from a whole genome. An analysis of *Helicobacter pylori* sigma(80) family of promoter signals**. *J Mol Biol* 2000, **297**:335-53.
30 Eskin E, Keich U, Gelfand MS, Pevzner PA: **Genome-wide analysis of bacterial promoter regions**. *Pac Symp Biocomput* 2003:29-40.
31 Mwangi MM, Siggia ED: **Genome wide identification of regulatory motifs in *Bacillus subtilis***. *BMC Bioinformatics* 2003, **4**:18.
32 Li H, Rhodius V, Gross C, Siggia ED: **Identification of the binding sites of regulatory proteins in bacterial genomes**. *Proc Natl Acad Sci U S A* 2002, **99**:11772-7.
33 Studholme DJ, Bentley SD, Kormanec J: **Bioinformatic identification of novel regulatory DNA sequence motifs in *Streptomyces coelicolor***. *BMC Microbiol* 2004, **4**:14.
39 Burden S, Lin YX, Zhang R: **Improving promoter prediction for the NNPP2.2 algorithm: a case study using *E-Coli* DNA sequences**. *Bioinformatics* 2004.
78 Reese MG: **Application of a time-delay neural network to promoter annotation in the *Drosophila melanogaster* genome**. *Comput Chem* 2001, **26**:51-6.
