# Supplementary material for: Detection of prokaryotic promoters from the genomic distribution of hexanucleotide pairs
Source: BMC Bioinformatics. 2006 Oct 2;7:423. doi: 10.1186/1471-2105-7-423 (PMC1615881; doi:10.1186/1471-2105-7-423)
Supplement: Additional file 6 — Comparison with normalized PSWM scoring function. Comparison of our integral method with a method in which the scoring function was replaced by normalized PSWM scores. [file 1471-2105-7-423-S6.pdf]

Additional file 6 – Comparison with normalized PSWM scoring function

		Organism-specialized mat.		General matrix		Normalized *E. coli* PSWM		Normalized *B. subtilis* PSWM	
**Organism**	**Promoters**	**Sensitivity**	**FP/100 nt**	**Sensitivity**	**FP/100 nt**	**Sensitivity**	**FP/100 nt**	**Sensitivity**	**FP/100 nt**
E. coli	377	42.4%	1.13	31.0%	1.09	24.1%	0.11	20.2%	0.09
B. subtilis	148	56.8%	0.99	50.0%	0.93	38.5%	0.13	35.1%	0.12
C. glutamicum	34	29.4%	1.36	14.7%	1.36	26.5%	0.17	23.5%	0.18
M. pneumoniae	30	43.3%	1.08	30.0%	1.07	26.7%	0.07	23.3%	0.07
M. tuberculosis	28	57.1%	0.74	50.0%	0.84	25.0%	0.17	32.1%	0.17
S. coelicolor	17	58.8%	1.42	47.1%	1.27	29.4%	0.21	29.4%	0.21
H. pylori	17	47.1%	0.53	35.3%	0.70	0.0%	0.04	5.9%	0.06
C. jejuni	14	42.9%	0.84	35.7%	0.77	7.1%	0.07	0.0%	0.10
B. japonicum	11	90.9%	0.73	90.9%	0.97	45.5%	0.17	45.5%	0.19
S. aureus	8	37.5%	0.59	37.5%	1.13	12.5%	0.05	12.5%	0.05
