# Supplementary material for: Detection of prokaryotic promoters from the genomic distribution of hexanucleotide pairs
Source: BMC Bioinformatics. 2006 Oct 2;7:423. doi: 10.1186/1471-2105-7-423 (PMC1615881; doi:10.1186/1471-2105-7-423)
Supplement: Additional file 8 — Primer sequences used for biochemical validations. Primer sequences used for biochemical validations. [file 1471-2105-7-423-S8.pdf]

Additional file 8 – Primer sequences used for biochemical validations

**Organism**	**Gene Name**	**Primer sequence**
E. coli	*yfgA*	TGGCTTCAGTATTCATTCGCT
E. coli	*ygfE*	TATCGACGGGTTGTGCAGACA
E. coli	*secE*	ATTCGCACTCATAAACCAACC
E. coli	*tag*	GTTCCATACTTTCCCTCGCTA
B. subtilis	*proB*	AAGGCAGGTTCGGCAGGTT
B. subtilis	*lmrA*	GGGTAGCTGCCGAAAGGATTT
M. bovis BCG	*rpsA*	TCCTCGCTAGAGCCTATGTCG
